# Burosumab, a Transformational Treatment in a Pediatric Patient With Cutaneous-Skeletal Hypophosphatemia Syndrome

**DOI:** 10.1210/jcemcr/luae184

**Published:** 2024-10-16

**Authors:** Paulo Cesar Alves da Silva, Vinicius Rene Giombelli, Fernando Henrique Galvão Tessaro

**Affiliations:** Department of Pediatric Endocrinology, Hospital Infantil Joana de Gusmão, Florianópolis 88025-301, Brazil; Department of Pediatric Endocrinology, Hospital Infantil Joana de Gusmão, Florianópolis 88025-301, Brazil; Ultragenyx Brasil Farmacêutica LTDA, Rua Josefina, 200 Andar 1 Conj 115, Vila Progresso, Guarulhos 07093-080, Brazil

**Keywords:** burosumab, conventional treatment, CSHS, fibroblast growth factor 23, hypophosphatemic rickets

## Abstract

Cutaneous-skeletal hypophosphatemia syndrome (CSHS) is a rare disorder characterized by the presence of melanocytic nevi, dysplastic cortical bony lesions, and fibroblast growth factor 23 (FGF23)-mediated hypophosphatemic rickets. Herein, we describe the diagnosis of an 8-year-old girl presenting with short stature, reduced lower limb mobility, and abnormal gait due to muscle weakness and constant pain in the legs. Biochemical parameters demonstrated hypophosphatemia, hyperphosphaturia, slight increase in parathyroid hormone (PTH), high levels of alkaline phosphatase, and elevated FGF23. Burosumab improved phosphate-wasting, serum phosphorus, alkaline phosphatase, and PTH, followed by a significant mineralization in vertebral bodies evidenced by radiographic assessment. Our report shows a long-term follow-up of CSHS with a notable improvement promoted by an anti-FGF23 therapy.

## Introduction

Cutaneous-skeletal hypophosphatemia syndrome (CSHS) is a rare disorder that affects primarily skin/bone with an existence of epidermal/melanocytic nevi, and an excessive production of fibroblast growth factor 23 (FGF23) [1, 2]. Increased levels of FGF23 impairs phosphate metabolism by reducing renal tubular reabsorption [3]. FGF23 also has a role in reducing renal 1α-hydroxylase activity and stimulating the expression of 24-hydroxylase leading to a lower conversion of the active form 1,25-dihydroxyvitamin D, which negatively affects the absorption of inorganic phosphate by the small intestine [4].

Patients with CSHS present with osseous manifestations such as bone pain, rickets, fractures, scoliosis, limb deformities, and impaired mobility [1]. Differential diagnoses of FGF23-mediated hypophosphatemia have been established, but the ideal clinical management of CSHS patients is still unclear [3]. Conventional therapy for CSHS patients consists of multiple daily doses of oral phosphate and vitamin D [1]. Limited benefits on clinical manifestation are seen in a small number of patients, despite side effects of gastrointestinal distress, hyperparathyroidism, hypercalcemia, and nephrocalcinosis [1, 3].

Burosumab is approved to treat patients with X-linked hypophosphatemia and tumor-induced osteomalacia [4, 5]. Safety and efficacy were reported in clinical studies as improvements in serum phosphate, alkaline phosphatase, and radiographic healing of rickets in children [5, 6]. Burosumab stands as an off-label recommendation to treat patients with CSHS [6-10]. Here, we describe a challenging diagnosis of a pediatric patient with CSHS during long-term use of burosumab after transitioning from conventional therapy.

## Case Presentation

**Figure 1.**
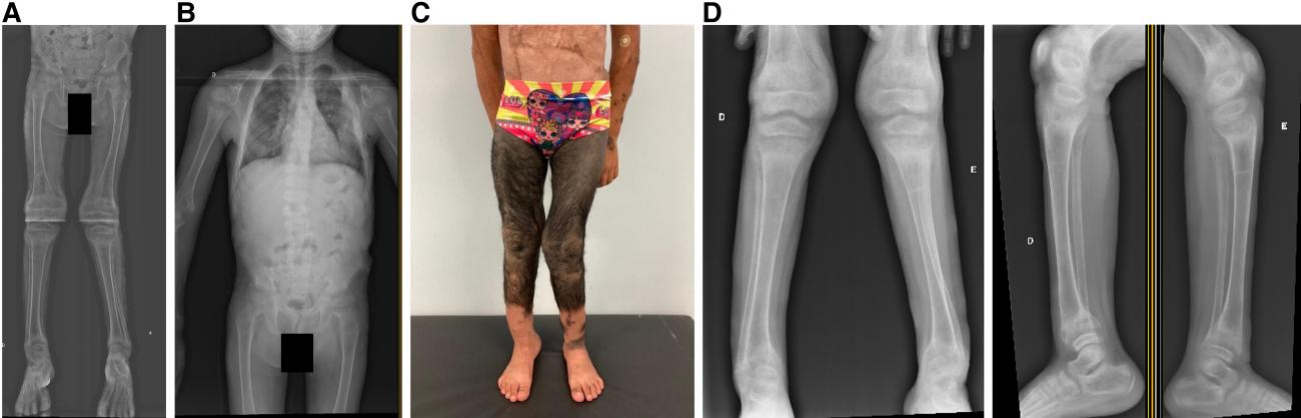
A, X-ray of the lower limbs, and B, panoramic x-ray of the spine. C, Patient presents with giant hairy nevus distributed on both legs and surgery scars in the abdomen region. D, Valgus knee and metaphyseal enlargement are shown in the radiographic images.

At age 1 year and 6 months, the patient was admitted by the pediatric service. Medical history revealed a preterm birth with a weight of 2.3 kg from a twin pregnancy of a nonconsanguineous marriage. The patient’s twin brother had normal stature and development. She exhibited a giant congenital melanocytic nevus covering both legs and another covering a substantial part of the dorsum, with smaller lesions spread across the body. The patient was referred to the orthopedic service due to short stature and delayed growth. She returned only at age 7 years; orthopedic evaluation revealed both knees exhibited a 30° angulation, while the right hip showed a 20° angulation and the left hip a 15° angulation (Fig. 1A), and her spine had an abnormal lateral curvature (Fig. 1B). Examination showed a more prominent genu valgum on the left side and positive to the Gowers sign. The initial suspicion was muscular dystrophy. Then, she was referred to the neurology and medical genetics teams for further medical investigation.

Neurological investigation showed that the patient exhibited normal serum levels of creatine kinase, aspartate transaminase, and aldolase, suggesting the absence of myopathy or hepatic dysfunction. Cognitive function was intact, and neurological assessment revealed intact cranial nerve function. Motor examination of the upper extremities demonstrated normal muscle strength, trophism, tone, and deep tendon reflexes. In contrast, the lower extremities showed decreased muscle trophism, particularly in the left thigh, diminished proximal muscle strength in the left lower extremity, and hypotonia. Hyperreflexia (++++) was noted, with a negative Babinski sign and an exhaustible Achilles clonus. Gait analysis revealed mild claudication and dragging of the lower extremities, accompanied by increased lumbar lordosis. These findings were consistent with paraparesis of the lower extremities and signs of pyramidal tract involvement. Back to the orthopedic clinics, magnetic resonance imaging presented a diffuse reduction with a loss of bone height in the vertebral body at level of thoracic (T)3, T6, T10, and T11 and lumbar (L)2 and L4 and absence of associated intradural alterations. Radiographs indicated signs of alterations in bone and mineral metabolism, and the patient was then referred to endocrinological evaluation at age 7 years and 7 months.

## Diagnostic Assessment

Serum analysis revealed hypophosphatemia of 1.7 ng/mL (0.5 mmol/L) (normal reference range, 3.30-5.20 mg/dL; 1.07-1.68 mmol/L); serum calcium levels were 9.7 mg/dL (2.42 mmol/L) (normal reference range, 8.8-10.7 mg/dL; 35.20-42.80 mmol/L), whereas parathyroid hormone (PTH) was slightly increased at 70.0 pg/mL (8.27 pmol/L) (normal reference range, 10.0-65.0 pg/mL; 1.06-6.9 pmol/L). Alkaline phosphatase was significantly increased to 4590 U/L (normal reference range <280 U/L), and 25-hydroxyvitamin D concentration was below normal at 12.6 ng/mL (31.4 nmol/L) (normal reference range, 30.0-60.0 ng/mL; 75.0-150.0 nmol/L). Height parameters were below the third percentile (1.01 m; *Z* score −4.96). A giant congenital melanocytic nevus was covering both of the patient’s legs with smaller lesions dispersed across the body (Fig. 1C). She was prepubertal. The patient started conventional therapy with oral calcitriol (1 × 0.25 mcg/day) and oral phosphate (3 × 60 mg/kg/day). Then, our team suspected hypophosphatemic rickets, including X-linked hypophosphatemia, vitamin D deficiency, or a tumor producing FGF23. Our radiologic investigation showed that at age 8 years, she presented with an impairment in bone mineralization (Fig. 1D and 1E). FGF23 evaluation showed abnormal high levels of 185 kRU/L in serum (normal reference range, 26-110 kRU/L). Genetic tests to evaluate RAS mutations in skin samples were not commercially available in our territory; therefore, the exclusion diagnosis of hereditary rickets was performed by family history only. Elevated FGF23 reduces the fractional tubular reabsorption of phosphate (TRP) and the renal tubular reabsorption of phosphate, commonly measured by the ratio of tubular maximum reabsorption of phosphate to glomerular filtration rate (TmP/GFR) [3, 4]. The TRP level was found to be 73%, and the TmP/GFR ratio was 1.60 mg/dL (0.52 mmol/L), with a normal reference range of 2.9 to 6.5 mg/dL (1.15-2.44 mmol/L).

**Figure 2.**
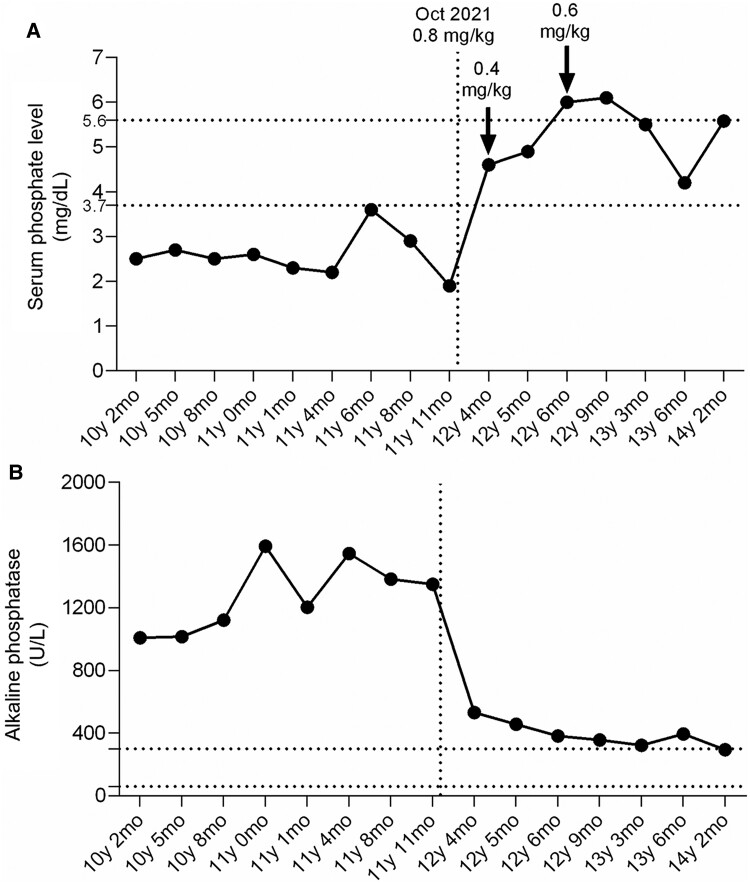
A, Serum phosphate and B, alkaline phosphatase change from baseline to the last evaluation (December 2023). Transitioning from conventional therapy to burosumab 0.8 mg/kg started October 2021 (vertical dotted line). Arrows indicate burosumab dose escalation. Reference value of serum phosphate (3.3-5.2 mg/dL) and alkaline phosphatase (below 280 U/L) levels are indicated in the horizontal dotted lines.

The extensive melanocytic nevi, impaired phosphate metabolism, and phosphate-wasting with an increase of FGF23, in addition to limb deformities and scoliosis, suggested the diagnosis to CSHS by age 8 years. With conventional therapy, the patient reported improvement (Fig. 2A and 2B), but treatment became a burden leading to issues with adherence to the treatment regimen. At age 8 years and 2 months, ultrasound examinations revealed no abnormalities in the parathyroid glands. Back to the neurology team, she was discharged from neurology because the assessment indicated that the child's gait abnormalities were due to bone and muscular issues. At age 10 years, an ultrasound of the kidneys and urinary tract was conducted to evaluate for possible nephrocalcinosis; this also showed no changes. In March 2021, at age 11 years and 7 months, a sestamibi whole-body scan did not identify any tumors. Our investigation was extended to understand if the natural history could alter the heart properties [1]. At age 12 years, the patient's electrocardiogram revealed a normal sinus rhythm. Cardiac axis was preserved, indicating normal electrical activity and alignment of the heart, and the corrected QT interval fell within the normal range. At age 14 years, additional genetic testing performed showed no abnormalities in renal phosphate-handling genes (*DMP1*, *FGF23*, *FGFR1*, *GALNT3*, *PHEX*, *SLC34A1*, *SLC34A3*, *SLC9A3R1*).

## Treatment

Burosumab was initiated when the patient was age 12 years at a starting dose of 0.8 mg/kg every 2 weeks (see Fig. 2).

## Outcome and Follow-Up

**Figure 3.**
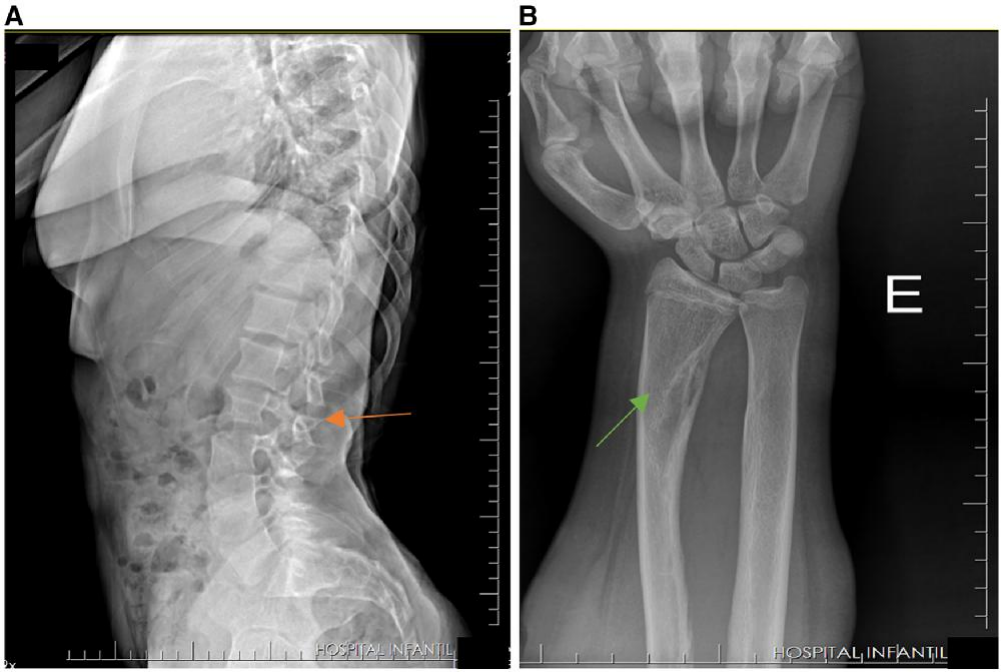
Radiographic images showing the patient aged 14 years and 10 months during use of burosumab. A, X-ray of the lumbar spine. Arrow indicates the first lumbar vertebra (L1), which is used as a reference point for assessing thoracolumbar dextroscoliosis; B, x-ray of the right wrist. Arrow indicates a cortical defect in the distal radius of the left wrist.

The dose of 0.8 mg/kg of burosumab caused hyperphosphatemia (Fig. 2A), and a reduction in alkaline phosphatase level was also evidenced (Fig. 2B). So, we adjusted burosumab to 0.4 mg/kg. Significant improvements in mobility and pain were reported by the patient and her parents during a few months of treatment. After 9 months of therapy (see Fig. 2A), the dose was readjusted to 0.6 mg/kg. Her laboratory test results showed that PTH, 1,25 dihydroxyvitamin D, and TmP/GFR were in the normal range; meanwhile serum phosphorus and alkaline phosphatase presented as slightly increased (Table 1). At age 14 years and 10 months, the patient exhibited vertebral bodies in anatomical alignment, preserved intervertebral disc spaces, and thoracolumbar dextroscoliosis (Fig. 3A); the left wrist showed a cortical defect in the distal radius possibly due to bone fragility and previous fracture not reported by the patient (Fig. 3B).

**Table 1.** Clinical characteristics, anthropometric measurements, and laboratory data at the diagnosis, pre-burosumab, and post-burosumab

Clinical characteristics				
Age at CSHS diagnosis, y	7.8			
Duration of conventional therapy, y	4.4			
Age at burosumab initiation, y	12			

Anthropometric measurements	March 2020 (10 y 5 mo/conventional therapy)	October 2021 (12 y/pre-burosumab)	December 2023 (14y 2 mo/burosumab)	Reference value*^a^*
Height, m	1.09	1.22	1.33	
Height *Z* score	−4.5	−4.5	−4.5	
Weight, kg	18.6	26.1	39.0	
BMI	15.7	17.7	22.2	
BMI *Z* score	−0.64	−0.14	0.90	
**Laboratory data Serum**				
Phosphorus	2.70 mg/dL (0.87 mmol/L)	1.90 mg/dL (0.61 mmol/L)	5.58 mg/dL (1.80 mmol/L)	3.30-5.20 mg/dL (1.07-1.68 mmol/L)
25-Hidroxyvitamin D	42.9 ng/mL (107.1 nmol/L)	20.3 ng/mL (50.7 nmol/L)	10.8 ng/mL (26.9 nmol/L)	30.0-60.0 ng/mL (75.0-150.0 nmol/L)
1,25-Dihydroxyvitamin D	—	—	88.7 pg/mL (212.9 pmol/L)	19.9-79.3 pg/mL (48.0-90.0 pmol/L)
PTH	67.7 pg/mL (7.18 pmol/L)	62.9 pg/mL (6.67 pmol/L)	40.6 pg/mL (4.31 pmol/L)	10.0-65.0 pg/mL (1.06-6.90 pmol/L)
Calcium	9.0 mg/dL (36.0 mmol/L)	9.0 mg/dL (36.0 mmol/L)	9.5 mg/dL (38.0 mmol/L)	8.8-10.7 mg/dL (35.2-42.8 mmol/L)
Alkaline Phosphatase	1016 U/L	1314 U/L	294 U/L	<280 U/L
Creatinine	0.3 mg/dL (26.5 µmol/L)	0.4 mg/dL (35.4 µmol/L)	0.4 mg/dL (34.5 µmol/L)	0.6-1.1 mg/dL (53.0-97.2 µmol/L)
**Laboratory data Urine**				
Phosphorus	87 mg/dL (28.09 mmol/L)	115 mg/dL (37.13 mmol/L)	219 mg/dL (70.72 mmol/L)	7-148 mg/dL (2.26-47.79 mmol/L)
Creatinine	27.8 mg/dL (2457.6 µmol/L)	111.0 mg/dL (9810.8 µmol/L)	178.0 mg/dL (15735.5 µmol/L)	16.0-327.0 mg/dL (1414.4-28907.3 µmol/L)
TRP	65%	78%	91%	85%-95%
TmP/GFR	1.76 mg/dL (0.57 mmol/L)	1.49 mg/dL (0.48 mmol/L)	5.58 mg/dL (1.65 mmol/L)	2.90-6.50 mg/dL (1.15-2.44 mmol/L)

Abbreviations: BMI, body mass index; CSHS, cutaneous-skeletal hypophosphatemia syndrome; PTH, parathyroid hormone; TmP/GFR, ratio of tubular maximum reabsorption of phosphate to glomerular filtration rate; TRP, tubular reabsorption of phosphate.

^*a*^Reference values were converted from conventional units to International System of Units.

## Discussion

CSHS was first described in 2016 [1]. The diagnosis predominantly relies on a thorough clinical evaluation and the identification of characteristic features. Key aspects of the diagnostic process include the assessment of epidermal and/or melanocytic nevi, skeletal dysplasia, fractures, limb deformities, and hypophosphatemia caused by elevated levels of FGF23. The underlying pathogenic event in CSHS has been identified as somatic mosaic mutations in the RAS genes. Genetic evaluation is therefore an important component of the diagnostic process.

Conventional treatment has been used to delay the consequences caused by chronic hypophosphatemia, but a small benefit is reported in the literature [5-10]. Another alternative approach used in the past was the surgical resection or laser ablation of small lesions, which failed to reduce the levels of FGF23 or improve the biochemical parameters [2, 11, 12]. Surgical resections were performed several times in our patient with no normalization of the phosphate serum levels or effect on her symptoms. Besides that, long-term use of conventional therapy is associated with incomplete healing of rickets, residual skeletal deformity, persistent short stature, gastrointestinal side effects, high risks of hypercalciuria, nephrocalcinosis, and hyperparathyroidism [13]. Direct effects on FGF23 are seen in CSHS as well as X-linked hypophosphatemia and tumor-induced osteomalacia by suppressing renal phosphate reabsorption. Burosumab targets FGF23, augmenting the low-normal phosphate levels, which requires periodic measurements at peak and trough time points [3, 5, 14]. Recently, case reports of patients with CSHS and McCune-Albright syndrome demonstrated the efficacy and safety of treating these syndromes with burosumab [7-9, 15, 16].

Treating patients with CSHS with burosumab has restored phosphate homoeostasis, leading to durable improvements in rickets, pain, and physical function [6-10]. Consistently, our case report shows that treatment increased serum phosphate levels by improving the TmP/GFR and 1,25 dihydroxyvitamin D, and reduced high levels of alkaline phosphatase, which has an important role in bone metabolism. The improvements in bone health specifically in the vertebral body at the thoracic and lumbar spine were largely attributed to the use of burosumab; however, these findings were revealed by different imaging techniques. Improvement in the quality of life has been reported both by the patient and her parents. She can walk and run better, with less school absenteeism, which promotes greater socialization at school and allows her to have a more active social life, as the pain is not as frequent as before. Currently, she is evaluated by pediatric endocrinology every 3 to 4 months and by dermatology annually. Our results underscore the importance of early diagnosis and tailored treatment plans in pediatric bone health management. Despite methodological limitations, our case report highlights the need for additional studies on the long-term effect of burosumab on the clinical manifestations of CSHS.

## Learning Points

- In the presence of giant skin lesions (nevus), muscle weakness, muscle and bone pain, fatigue, short stature, lower limb deformity in pediatric patients, serum phosphate, TmP/GFR, and alkaline phosphatase should be measured and CSHS considered.
- Neutralizing the high levels of FGF23 improved phosphate metabolism by increasing the phosphate levels in serum and the TmP/GFR and decreasing the levels of alkaline phosphatase over the course of the treatment.
- Burosumab is a potential therapeutic option to manage hypophosphatemia related to FGF23 in patients with CSHS.

## Data Availability

Original data generated and analyzed during this study are included in this published article.

## Abbreviations

BMI: body mass index
CSHS: cutaneous-skeletal hypophosphatemia syndrome
FGF23: fibroblast growth factor 23
L: lumbar
PTH: parathyroid hormone
T: thoracic
TmP/GFR: ratio of tubular maximum reabsorption of phosphate to glomerular filtration rate
TRP: tubular reabsorption of phosphate
